# Supplementary figures and images for: Stable dominance of parasitic dinoflagellates in Antarctic sponges
Source: PeerJ. 2024 Nov 8;12:e18365. doi: 10.7717/peerj.18365 (PMC11552495; doi:10.7717/peerj.18365)

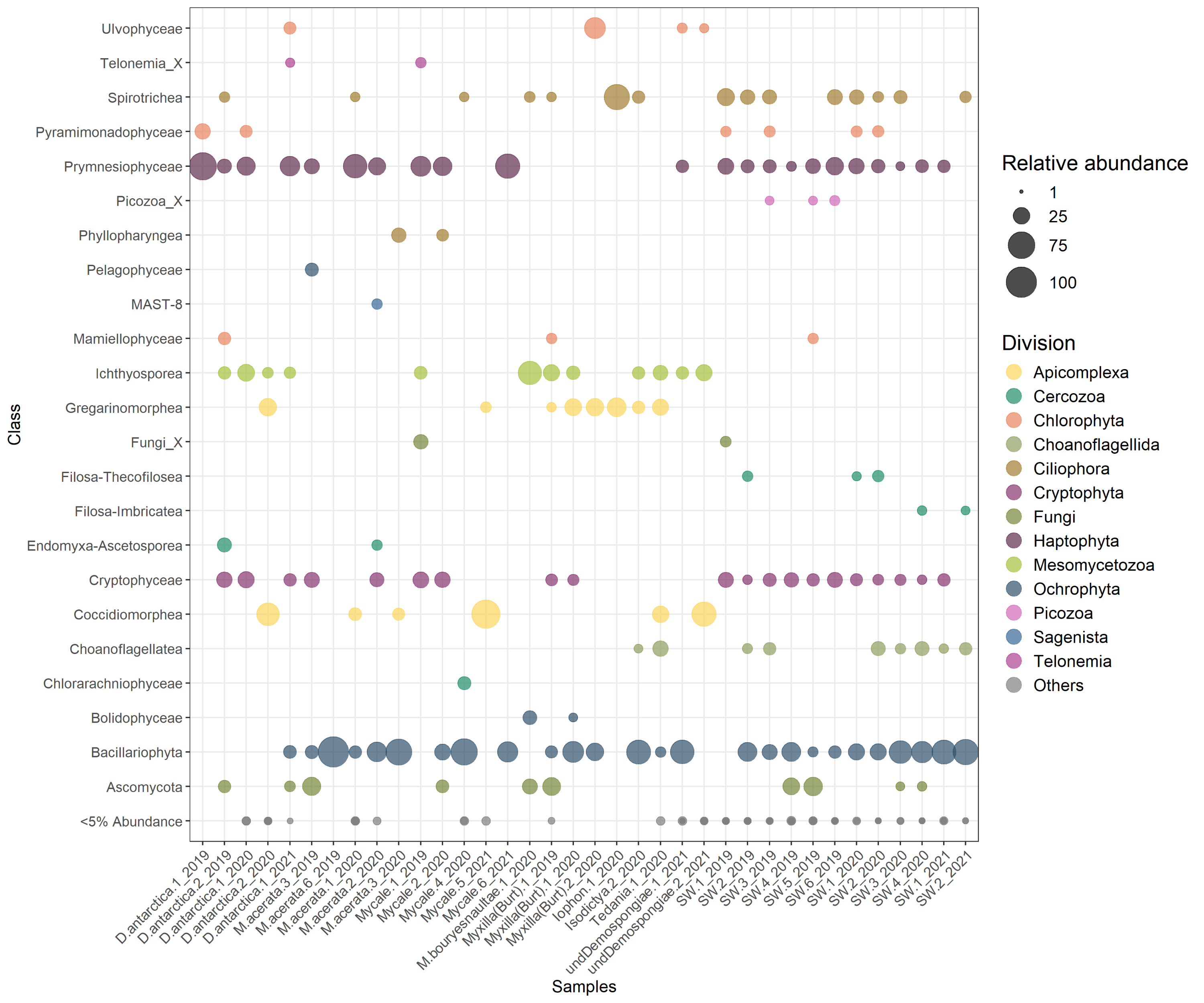

Supplement: Supplemental Information 1 — Only classes with a relative abundance above 5% are displayed; those below this threshold are categorized as <5% Abundance. The bubble colors represent the division level of the non-dino community, according to the PR2 database. Sample names are denoted using the sponge species, replicate and summer of sampling. D.antarctica: Dendrilla antarctica; M.acerata: Mycale acerata; M.bouryesnaultae: Mycale bouryesnaultae; Myxilla(Burt): Myxilla (Burtonanchora) sp.; undeDemospongiae: undetermined Demospongiae; SW: surrounding seawater. [file peerj-12-18365-s001.png]

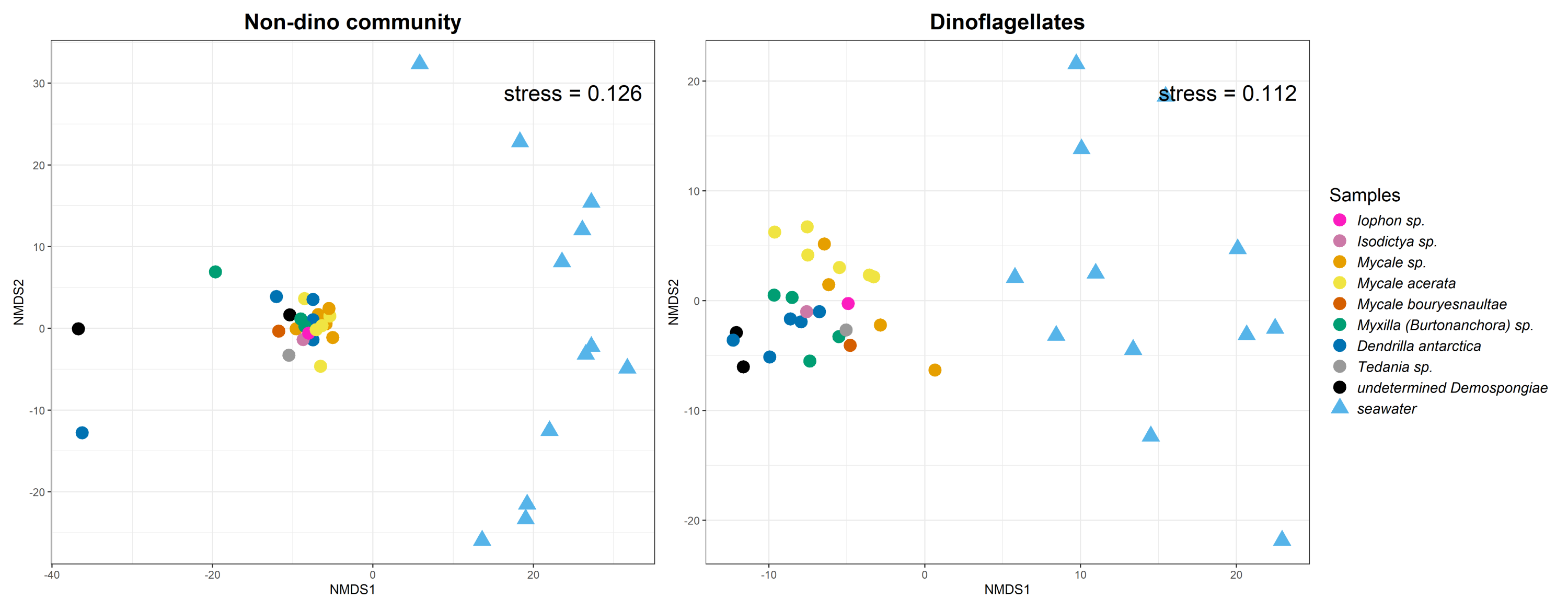

Supplement: Supplemental Information 2 — Based on centered log-ratio-transformed Aitchison distances matrices of the ASV relative abundances, the analysis is shown for the non-dino community (left panel) and dinoflagellates (right panel). Analysis of Similarities (ANOSIM) was employed to test for differences in beta diversity between habitats (sponges and SW), using 999 permutations. The beta diversities of the non-dino community and dinoflagellates across sample types were significant (ANOSIM; p =0.001). ASVs with an abundance lower than 0.1% were removed. [file peerj-12-18365-s002.png]

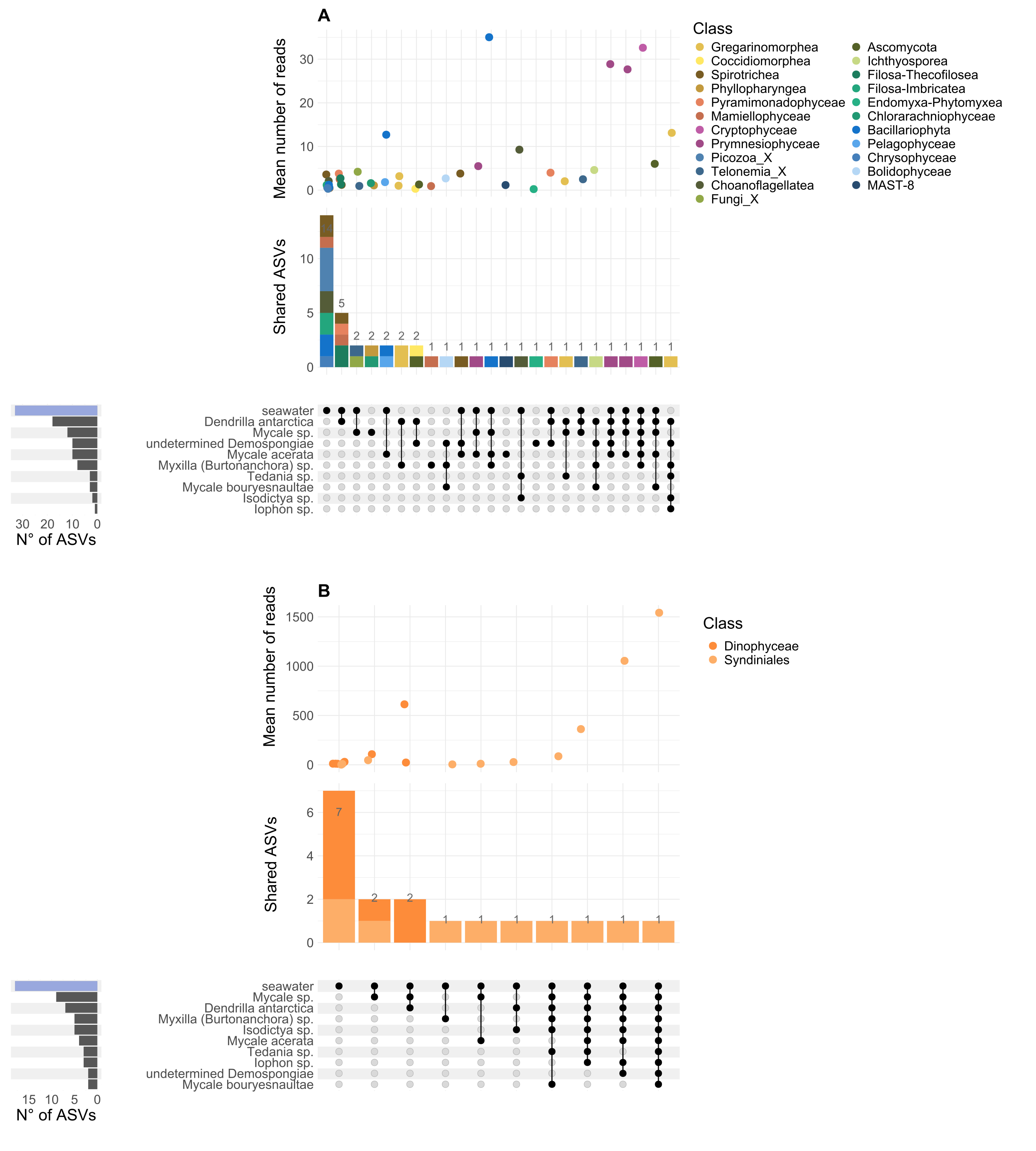

Supplement: Supplemental Information 3 — The UpSet plot shows the number of ASVs unique or shared between sponge species and SW for the non-dino community (A) and dinoflagellates (B). Colors represent the class of each ASV; mean read abundance is indicated at the top of each intersection. The bar chart on the left shows the number of ASVs for each group. [file peerj-12-18365-s003.png]

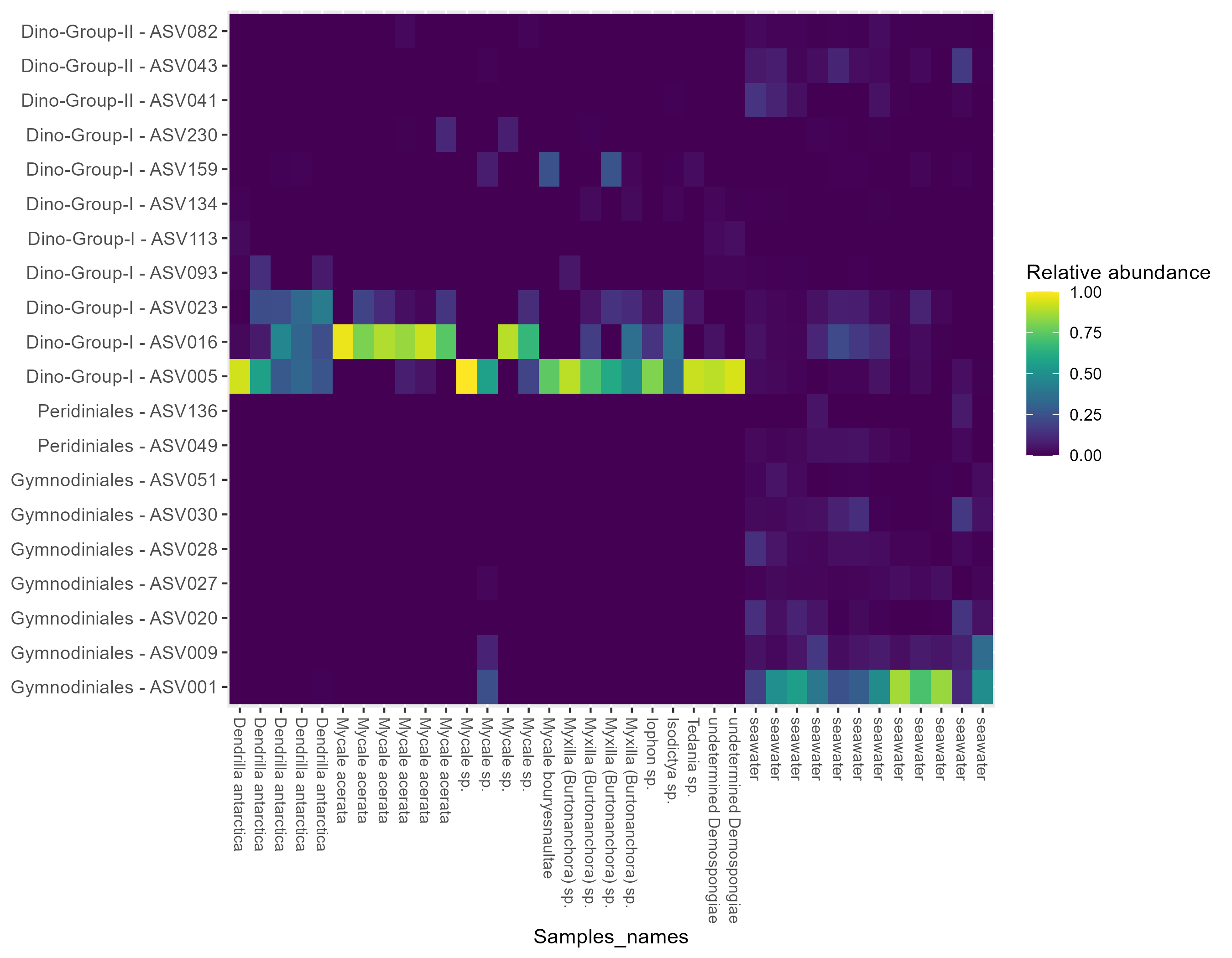

Supplement: Supplemental Information 4 — ASVs and their taxonomic classification at the order level according to the PR2 database are presented on the y-axis. The x-axis shows all samples. Analyses were conducted on ASVs representing the top 90% of the most abundant sequences in at least one sample. [file peerj-12-18365-s004.png]

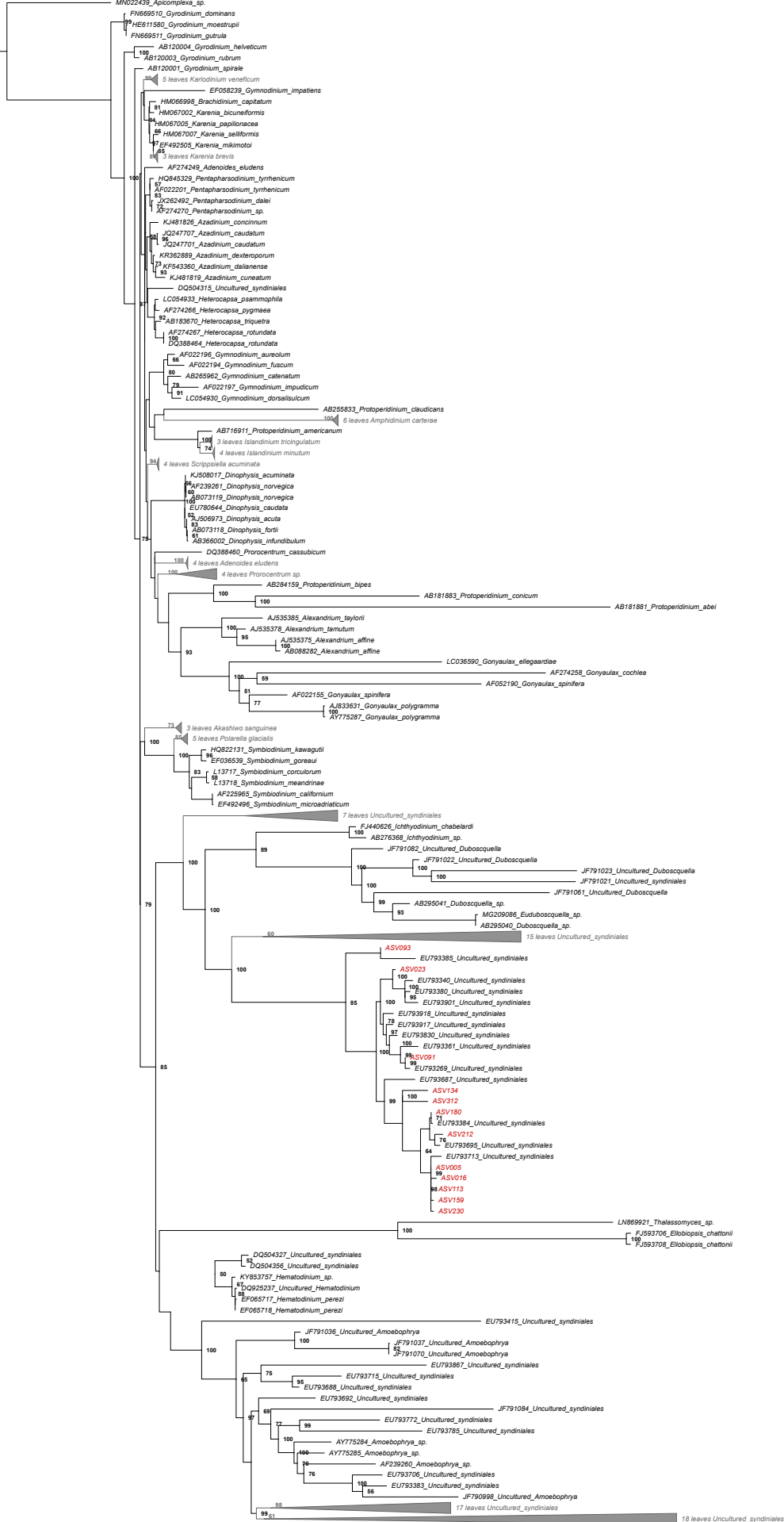

Supplement: Supplemental Information 5 — The RAxML evolutionary placement algorithm (EPA) placed short reads of Dino-Group-I-Clade 1 ASVs (in red) into the reference tree. The reference tree was constructed from an alignment of 211 sequences with 1,734 characters, with bootstrap support calculated from 100 replicates. Only bootstrap support values greater than 50% (out of 100) are shown. [file peerj-12-18365-s005.pdf]
